# Distinctive spinal changes in two patients with unusual forms of autosomal dominant endosteal hyperostosis: a case series

**DOI:** 10.1186/1752-1947-1-142

**Published:** 2007-11-22

**Authors:** Ali Al Kaissi, Franz Varga, Shahin Zandieh, Klaus Klaushofer, Franz Grill

**Affiliations:** 1 Ludwig Boltzmann Institute of Osteology at the Hanusch Hospital of WGKK and AUVA Trauma Centre Meidling, 4th Medical Department, Hanusch Hospital, Heinrich Collin Str. 30 A-1140, Vienna, Austria; 2 Radiology department, at Hanusch Hospital. Heinrich Collin Strasse. 30 A-1140, Vienna, Austria; 3 Orthopaedic Hospital of Speising, Speisinger Strasse. 109, Vienna-1130, Austria

## Abstract

Endosteal hyperostosis was encountered in a 26-year-old-man and his 6-month-old daughter. Both the father and his daughter presented with fractures. Odontoid process hyperplasia, and progressive sclerosis of the posterior spinal elements, was the other significant features. To the best of our knowledge, this is the first clinical report describing distinctive spinal changes in association with fractures and endosteal hyperostosis.

## Introduction

In 1966, Worth and Wollin described a condition of hyperostosis corticalis generalisata which was dominantly inherited [1]. It is radiologically similar to the autosomal recessive condition called van Buchem syndrome, and some authors refer to the two conditions as endosteal hyperostosis [2]. Facial dysmorphism and diaphyseal radiographic changes are present by adolescence and consist of an elongation of the mandible and an increased gonial angle. The forehead becomes flattened and there is a slowly enlarging osseous prominence of the hard palate (torus palatinus). The early radiographic changes include thickening of the endosteum of the long bones and the skull. A progressive increase in the density of the posterior elements of the spine has been noted in some patients [3-5].

More than 13 kindreds with endosteal hyperostosis have been reported. Four families out of 13 had autosomal dominant inheritance, including male-to-male transmission. Only two patients have been reported in the literature with a history of fractures [6].

We present a father and daughter with endosteal hyperostosis. They were unusual in that both had fractures, odontoid hyperplasia (subclinical basilar invagination), and a simultaneous process of anterior longitudinal spinal sclerosis along the thoracic vertebrae, associated with progressive sclerosis of the posterior elements of the spine. We found no previous reports describing this constellation of spinal abnormalities in association with autosomal dominant, endosteal hyperostosis.

## Case presentation

### Patient 1

A 6-month-old-girl was referred to the department of paediatric orthopaedics because of a fracture of the right humerus. The child was born at full term, the product of an uneventful gestation. At birth her weight, length and head circumference were around the 50^th ^percentile. Her mother was a 25-year-old gravida 1, abortus 0, married to a 26-year-old-unrelated father.

**Figure 1:**
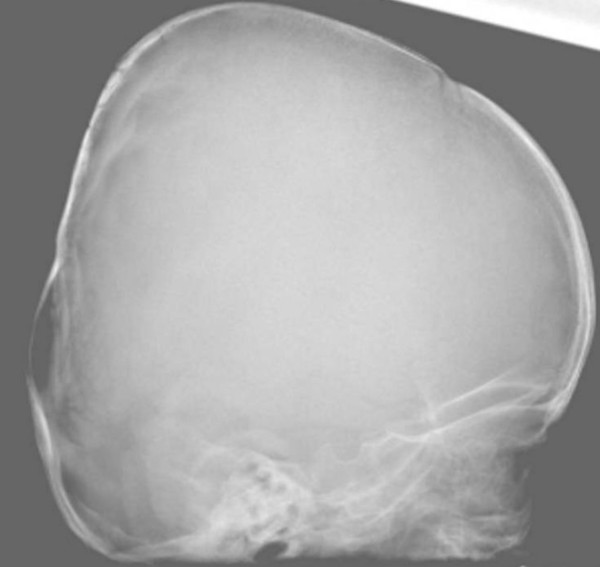
**(patient1)**. A lateral radiogram showed extensive flattening across the posterior skull and marked vault and convolutional sclerosis.

**Figure 2:**
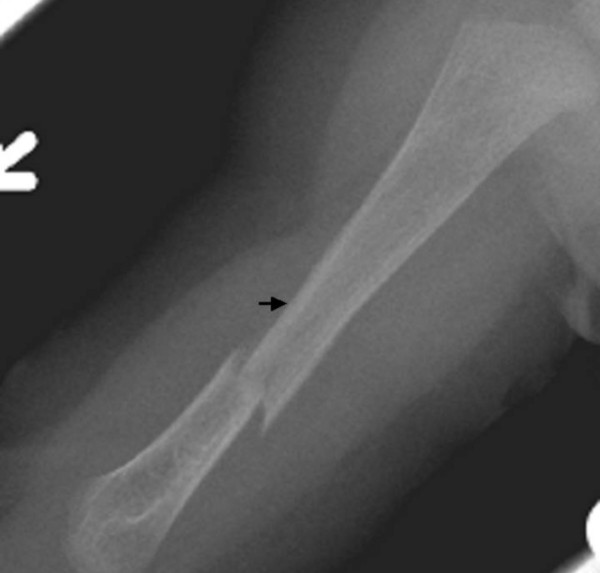
**(patient 1)**. An anteroposterior radiograph of the right humerus showed mid-diaphyseal fracture and unusual diaphyseal endosteal hyperostosis (arrow).

**Figure 3:**
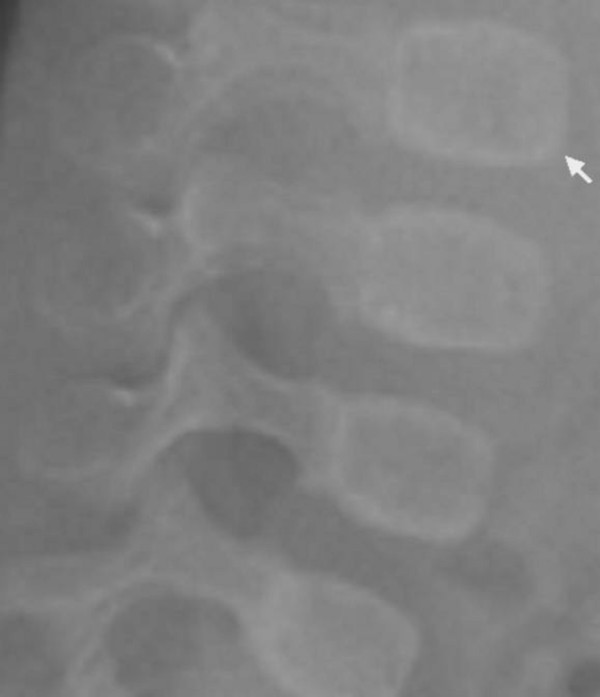
**(patient 1)**. The lateral spine radiograph showed no platyspondyly, but marked sclerosis of the entire vertebral cortical rim (arrow) and unusual enlargement of the spinous processes.

The child's development was normal and there was no history of serious illnesses. Clinical examination showed extensive flattening of the posterior aspect of the skull, which was brachycephalic. She had a flat face with mild frontal bossing, small and deeply set eyes, and low-set ears. Musculo-skeletal examination showed normal musculature and no associated anomalies. Blood biochemistry was normal. A lateral skull x-ray showed extensive flattening across the posterior skull and marked vault and convolutional sclerosis (figure 1). An AP radiograph of the right humerus showed mid-diaphyseal endosteal hyperostosis (arrow) and fracture (figure 2). The lateral spine radiograph showed no platyspondyly, but marked sclerosis of the entire vertebral rim circumference and unusual enlargement of the spinous processes (figure 3).

### Patient 2

A 26-year-old patient, the father of patient I, had a total of six fractures from early childhood till the preadolescent period, involving the clavicles and humerus, but none of the lower limb bones. Thereafter no fractures were reported. Clinical examination showed a man of normal height and normal phenotype. He had normal sclera, teeth and hearing. He had ligamentous stiffness but no muscle wasting or myopathic features. Rigidity over the vertebral column, particularly over his kyphotic thoracic spine was notable. His limbs were not bowed.

**Figure 4:**
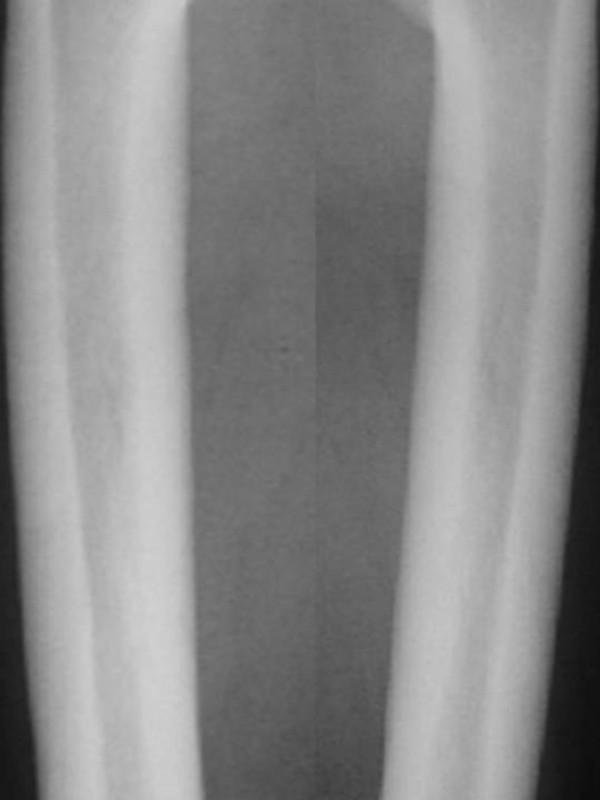
**(patient 2)**. Radiographic examination of the femora showed thick, and dense, endosteal hyperostosis more marked at the mid-diaphyses. The medullary canals were narrow but patent, and the endosteal surface was irregular (arrow).

**Figure 5:**
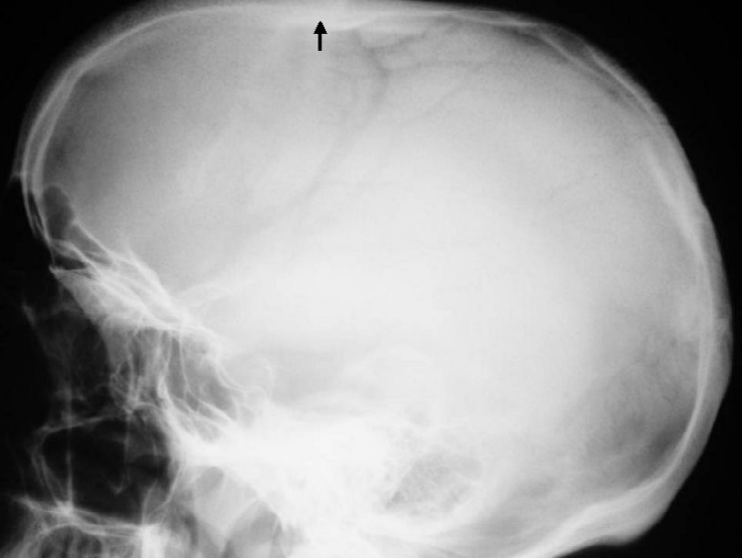
(patient 2). Lateral radiogram of the skull showed thick and sclerosed skull vault (arrow).

**Figure 6:**
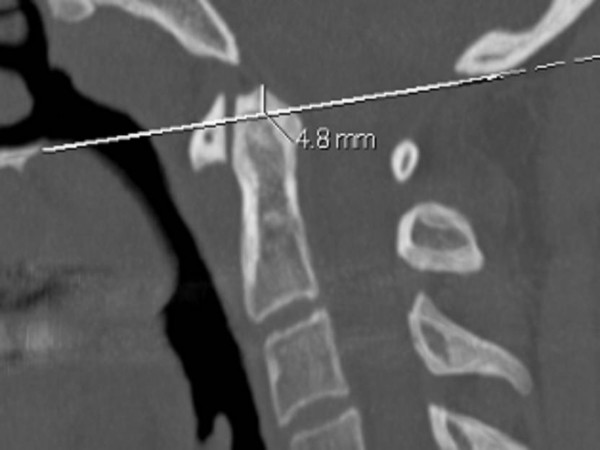
**(patient 2) **Sagittal reformatted multiplanar computed tomography of the craniocervical junction showed a hyperplastic odontoid process. The tip of the dens projected 4.8 mm above a line joining the back of the hard palate to the lowest point of the occipital squama (McGregor line). A mild basilar invagination was therefore present. Note the increased bone density of the posterior vertebral elements.

Radiographic examination of the femora showed thick, and dense, endosteal hyperostosis more marked at the mid-diaphyses. The medullary canals were narrow but patent, and the endosteal surface was irregular (figure 4). Lateral radiogram of the skull shows thick and sclerosed skull vault-arrow- (fig 5). Sagittal reformatted multiplanar computed tomography of the craniocervical junction showed a hyperplastic odontoid process. The tip of the dens projected 4.8 mm above a line joining the back of the hard palate to the lowest point of the occipital squama (McGregor line). Subclinical basilar invagination was therefore present. Note the increased bone density of the posterior vertebral elements (figure 6).

**Figure 7:**
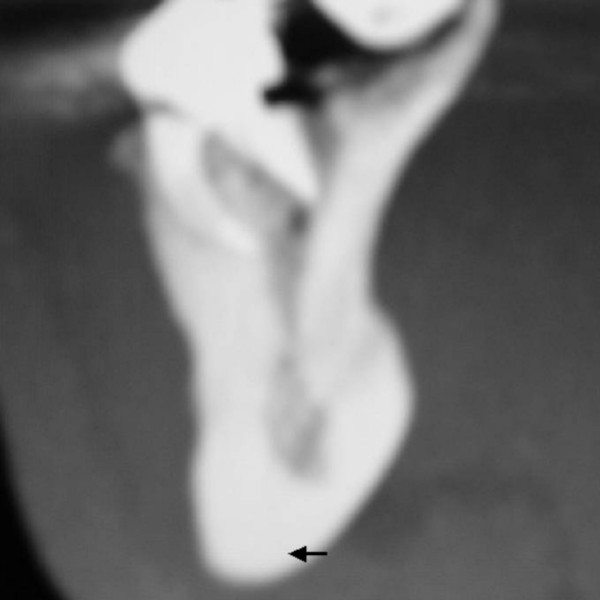
**(patient 2)**. Sagittal reformatted multiplanar computed tomography showed irregular increased density in the mandibular bone and the increased density of cancellous bone at the inferior borders.

**Figure 8:**
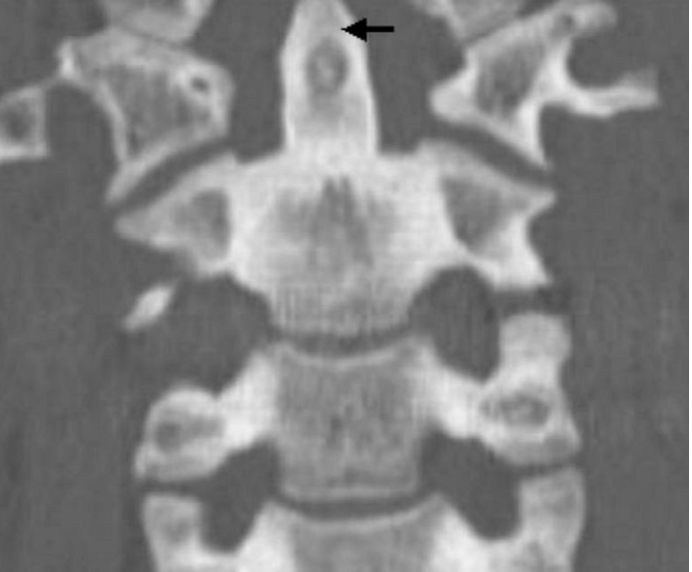
**(patient 2)**. Coronal reformatted multiplanar computed tomography of the cervical spine, demonstrating the increased density of the transverse processes and the unduly long odontoid process.

**Figure 9:**
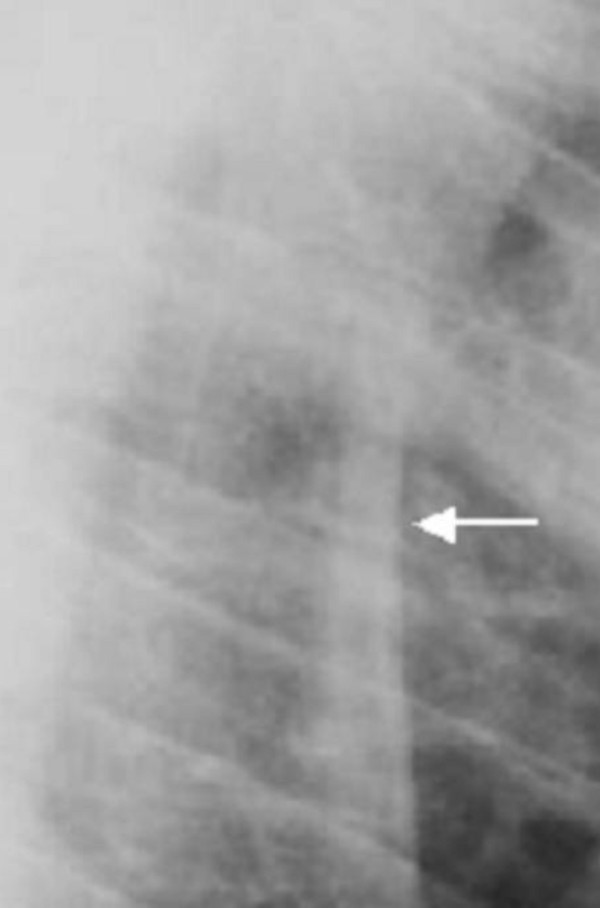
**(patient 2)**. Lateral radiograph of the thoracic vertebrae showed progressive sclerosis of the anterior longitudinal ligaments with subsequent development of bony ankylosis (arrow).

Sagittal reformatted multiplanar computed tomography demonstrated irregular increased density in the mandibular bone and the increased density of cancellous bone at the inferior borders (figure 7-arrow). Coronal reformatted multiplanar computed tomography of the cervical spine demonstrating the increased density of the transverse processed and the unduly long odontoid process-arrow- (fig 8). Lateral radiograph of the thoracic spine showed progressive sclerosis of the anterior longitudinal ligament with subsequent development of bony ankylosis (arrow-fig 9).

We measured the bone density in the hip and spine by means of central DEXA (Dual-energy x-ray absorptiometry). Lumbar spine showed osteoporosis (T-Score -2.9 SD), whereas the hip showed normal limits (T-Score -1.0SD).

## Discussion

Hyperostosis corticalis generalisata refers to a group of sclerosing bone diseases, the principal feature of which is a generalised, symmetrical, endosteal sclerosis at different sites of intramembraneous ossification. Four types of endosteal hyperostosis (van Buchem disease, Worth disease, Nakamura disease, and Truswell-Hansen disease) have been reported.

These differ from each other on the basis of the mode of inheritance and the involvement of the cranial nerves. Van Buchem's disease, for instance, is an autosomal recessive disease whose main features include involvement of the mandible, causing a misshapen jaw, unlike the relative normal facial phenotype seen in our patient [1-9].

Worth and Wollin, first described an autosomal dominant endosteal hyperostosis [1], in which early radiographic changes included thickening of the endosteum of the long bones and of the skull. Classically, the affected bones in patients with endosteal hyperostosis are resistant to fracture and only two patients with a fracture have been reported [6]. Our patients were unusual in that both presented with multiple fractures.

Spinal changes have rarely been reported in the Worth type of endosteal sclerosis. Perez-Vicente *et al*., [10] reported a father and daughter, with endosteal hyperostosis. Both showed severe sensorineural hearing loss, chronic intracranial hypertension, and mild corticospinal tract involvement. A cranial CT scan documented a reduction in size of the posterior fossa with encroachment at the foramen magnum. Neither fractures nor distinctive spinal changes were documented. Ades *et al*., [5] described the gradual increase in density of the posterior elements of the spine, which may be associated with arthritis and may lead to nerve entrapment.

Camurati-Engelmann disease (CED) [11], was considered. But, in our patient, the absence of muscle weakness, fatigue, poor appetite, headache and pain in the limbs, important features encountered in CED, were absent. Moreover, in CED the long bones are thickened and bone prominences and tenderness occur. These were not present in our patient.

Malformations of the craniocervical junction have been reported in patients with bone fragility. They have been found in association with osteomalacia, Paget's disease, hyperparathyroidism, rheumatoid arthritis, and osteogenesis imperfecta. In osteogenesis imperfecta, basilar impression rather than basilar invagination, is the rule [12]. Our patient had hyperplasia of the odontoid. This is defined as hypertrophy of the apical portion of the odontoid process of the axis. It can occur in Marfan syndrome, where height acceleration is a prominent feature and dolichoodontoid can cause sudden death [13]. Neither the phenotype, nor the radiographic features in our patient, was in favour of Marfan syndrome.

Our patient had a progressive anterior and posterior vertebral hyperostosis in connection with progressive sclerosis. The pattern was irrelevant to the diffuse idiopathic skeletal hyperostosis syndrome (DISH). The latter is observed in elderly patients with a history of disturbed metabolic parameters [14]. In our patient there is thoracic spine ankylosis involved the anterior and the posterior spine elements simultaneously; in a way it gave no chance to maintain skip areas of ankylosis. Spinal segments ankylosis might be a major predisposing factor for the development of marked spinal osteopenia and possibly osteoporosis and fracture [15]. Minor trauma can lead to serious fracture of the ankylosed spine.

## Conclusion

We present an unusual father-daughter pair with endosteal hyperostosis not compatible with Worth syndrome, spinal changes and multiple fractures. Although we have not been able to elucidate the actual pathogenesis in our patients, it sounds the likelihood that the disorder results from a simultaneous uncoupling of formation and resorption.

We suggest that the constellation of spinal changes seen in our patient was possibly caused by progressive prenatal, axial and extra-axial sclerosis. The fact of decreased bone density in the father could possibly correspond with the disturbed postnatal osteoblast-mediated bone formation to defective osteoclastic bone resorption.

On the other hand, the craniocervical changes were important to detect, as subclinical basilar invagination has the potential for severe neurological consequences. Anterior bony ankylosis over the thoracic spine is an additional risk for the development of myelopathy and fractures and nerve entrapment might result from the progressive involvement of the sclerotic posterior elements of the spine. Finally we wish to stress, that in the light of incompatibility between our current family and other forms of well-documented endosteal hyperostosis, the overall clinical and radiographic phenotype were distinctive and possibly represent a new syndrome.

## Competing interests

The author(s) declare that they have no competing interests.

## Authors' contributions

All authors read and approved the final manuscript and all participate in this work
